# Exploring Pragmatic Factors on the Logical Relationships of Conditional Reasoning: A Study of Counterfactual and Hypothetical Conditionals

**DOI:** 10.3390/bs14080686

**Published:** 2024-08-08

**Authors:** Lingda Kong, Yanting Sun, Xiaoming Jiang

**Affiliations:** 1 Institute of Corpus Studies & Applications, Shanghai International Studies University, Shanghai 201620, China; konglingda@shisu.edu.cn (L.K.); sunyanting@shisu.edu.cn (Y.S.); 2 Institute of Linguistics and Key Laboratory of Language Sciences and Multilingual Artificial Intelligence, Shanghai International Studies University, Shanghai 201620, China

**Keywords:** mental models, counterfactuals, hypothetical, temporal indicator, negator

## Abstract

Previous theories have established the mental model activation of processing different types of conditionals, stating that counterfactual conditionals expressing events that contradict known facts (e.g., “If it had rained, then they would not go to the park.”) are considered to trigger two mental models: (1) a hypothetical but factually wrong model (e.g., “rain” and “did not go to the park”) and (2) a corresponding real-world model (e.g., “did not rain” and “went to the park”). This study aimed to investigate whether pragmatic factors differentially influence readers’ comprehension and distinction between counterfactual and hypothetical conditional sentences in Mandarin Chinese. Participants were required to read and judge the comprehensibility of Chinese hypothetical and counterfactual conditionals, which were different in temporal indicators (past vs. future temporal indicators) in the antecedent. Different polarities (with vs. without negators) and different moving directions (different directional verbs: *lai2* [come] vs. *qu4* [go]) in the consequent were also manipulated. Linear mixed-effects models (LMEM) revealed that hypothetical conditionals (with future temporal indicators) were more comprehensible than counterfactual conditionals (with past temporal indicators). The semantic similarities within the subordinate clause revealed future temporal indicators had higher lexical–semantic co-occurrence than past indicators, suggesting that temporal indicators impact comprehension partly through lexical semantics in the premise, and hypothetical conditionals are more easily processed. However, the semantic similarity analysis of the main and the subordinate clauses showed no effect of temporal indicators, suggesting that lexical–semantic co-occurrence across clauses may not substantially contribute to the distinction between hypothetical conditionals and counterfactual conditionals. In conclusion, this study offers insights into the comprehension of Chinese conditional sentences by shedding light on the pragmatic factors influencing the activation of different mental models.

## 1. Introduction

In the realm of cognitive reasoning, mental model theory, notably advanced by Johnson-Laird, P.N. and Byrne, R.M.J. [1], presents a compelling perspective on the distinction between counterfactual and hypothetical conditional constructions. The mental model theory posits that individuals construct two distinct mental representations when encountering counterfactuals, for example, “If it had rained, then the street would be wet”: one representing the hypothetical event (i.e. rain and wet street) and the other implying the factual alternative (i.e. no rain and dry street) [2,3,4,5]. This dual representation aligns with two key constraints that define counterfactual conditionals: antecedent falsity and an implied causal relationship between the antecedent and consequent [6,7,8,9]. Conversely, hypothetical conditionals like “If it rains, then the street will be wet”, activate solely the hypothetical model of a possible world (i.e. rain and wet street) without explicitly addressing factual events [7]. This single representation primarily focuses on the implied causal relationship without necessitating the constraint of antecedent falsity. The distinction in mental model construction between counterfactual and hypothetical conditionals reflects the differential constraints governing these conditional types. Behavioral studies have consistently supported this differentiation, revealing distinct reasoning patterns and implications associated with counterfactual and hypothetical conditionals [10,11,12]. However, a significant research gap remains concerning whether pragmatic factors differentially influence the activation of single or dual mental models. This issue is particularly salient when distinguishing between counterfactual and hypothetical conditionals. Thus, this study aims to investigate whether pragmatic factors differentially influence readers’ comprehension and distinction between counterfactual and hypothetical conditional sentences in Mandarin Chinese. By focusing on this question, we sought to elucidate how various linguistic cues contribute to the processing of the constraints of antecedent falsity and implied causal relationships in sentence comprehension.

### 1.1. Multiple Pragmatic Factors Impact the Chinese Counterfactual and Hypothetical Conditionals

The fundamental difference in constraints highlights that the key to distinguishing between counterfactual and hypothetical conditionals lies in the presence and strength of antecedent falsity [13]. A critical question in the field of psycholinguistics is the following: What factors influence the strength of antecedent falsity in conditional statements? In Indo-European languages, the activation of the antecedent falsity depends on the morphosyntactic markers, such as the modal terms (e.g., could, might), morphological variations of past tense, and logical operator (e.g., if–then constructions) [14,15]. Behavioral studies have examined the acceptance rate of counterfactual conditionals (past tense and subjunctive mood) and hypothetical conditionals (past tense and indicative mood). These studies have generally shown that counterfactual conditionals tend to be accepted less often than hypothetical conditionals, providing suggestive evidence that counterfactuals imply scenarios with lower probability in the real world [11,16,17,18]. These morphosyntactic markers are explicit cues for antecedent falsity, facilitating the differentiation between counterfactual and hypothetical conditionals. However, regarding the various kinds of conditionals in Mandarin Chinese, Comrie, B. [19] and Donna, L. [20] indicated that unlike in Indo-European languages, no explicit morphological cues (such as the grammatical rules contingent on the subjunctive mood) exist in Chinese to distinguish between different kinds of conditional sentences. Instead, identifying Chinese counterfactual conditionals relies more heavily on lexical-pragmatic factors. These factors potentially influence the strength of antecedent falsity, including temporal indicators, directional verbs, and the consistency of described events with world knowledge [20].

Existing studies such as that by Yong, Q. [21] have examined the relationships between lexical-pragmatic factors and counterfactual versus hypothetical meaning through a large-scale corpus analysis of Chinese counterfactuals. To systematically identify counterfactuals within the corpus, Yong first conducted targeted searches for thirteen hypothetical conjunctions (e.g., *ruguo*, *tangtuo*, *jiaru*, and *yaoshi*) commonly used to mark counterfactuality in Chinese and then manually checked every example sentence to decide whether the statement expressed a counterfactual or a hypothetical meaning based on the factuality of context. For instance, “*ruguo youyike hongdou songgei ta jiuhaole*” [“If only I could give her a red bean”] (square bracket contains the English translation) could be considered as a hypothetical conditional since the factuality is not clear. In addition, the calculation of the co-occurrence of these factors in the corpus showed that Chinese counterfactuals were positively associated with the existence of negators (i.e., *meiyou* [there is not]) and past temporal indicators (i.e., *zuotian* [yesterday]). In the view of theoretical linguistics, Jiang, Y. [22] also proposed that using certain lexical features like hypothetical conjunction words (e.g., *yaobushi* [if it were not for] and *zaozhidao* [had known it earlier]), a temporal indicator or negator could help to strengthen counterfactual interpretation. 

The consistency of described events with world knowledge is a factor that may significantly impact the strength of antecedent falsity. When an event described in the antecedent contradicts established world knowledge, it might strongly signal antecedent falsity, thereby potentially activating counterfactual comprehension [17,19]. Consider the following sentence: “If the sun did not rise this morning, it would be dark outside”. Here, the phrase “the sun did not rise” violates our world knowledge, as it contradicts our daily experience. This violation of real-world knowledge assists readers in integrating and understanding the counterfactual nature of the statement. In Mandarin Chinese, constructions involving *bu* (no) and *meiyou* (there is not) can relate to such violations [17,20]. However, there is limited evidence regarding whether the consistency of described events with world knowledge differentially impacts the comprehension of counterfactual conditionals versus hypothetical conditionals. Whether readers process this type of world knowledge violation differently in counterfactual contexts than hypothetical ones remains unclear.

**Example** **1.** *“Ruguo jintian taiyang meiyou shengqi, waimian jiuhuishi heiande”.* *(“If the sun did not rise this morning, it would be dark outside”.)*

Temporal indicators play a crucial role in signaling antecedent falsity in Chinese counterfactuals, which refers to linguistic elements that specify the time frame of events described in conditional sentences. Temporal indicators enable readers to locate the time of the described event to make the timing of events explicit. Previous theoretical studies have regarded temporal indicators (e.g., *zuotian* [yesterday] and *mingtian* [tomorrow]) as the most basic lexical means of expressing hypothetical or counterfactual meaning in Chinese conditionals [23,24], and they are an important device in encoding a low-probability event in the antecedent of conditional sentences [21,22,23]. Yong, Q. [21] compared the frequency of different temporal indicators in counterfactual and non-counterfactual conditionals in a corpus, revealing that temporal indicators that describe the past are more frequently used in Chinese counterfactuals. On the contrary, a future temporal indicator (e.g., *mingtian* [yesterday]) is often be used in hypothetical conditionals [19,25] (see Example 3). However, there is still no direct empirical evidence to investigate the role of the temporal indicator in different conditional sentences. Thus, it is still unclear whether the involvement of a temporal indicator of the future makes it more difficult to construct hypothetical conditionals than does the temporal indicator that describes the past in counterfactual conditionals.

**Example** **2.** *“* *Ruguo zuotian buxiayu, xiaowang jiu lai tiqiu le”. (“If it had not rained yesterday, Xiaowang would have come to play soccer”.)*

**Example** **3.** *“* *Ruguo mingtian buxiayu, xiaowang jiu qu tiqiu le”. (“If it rains tomorrow, Xiaowang will go to play soccer”.)*

Thirdly, studies have shown directional verbs like *lai* (come) and *qu* (go) can also contribute to establishing antecedent falsity in Chinese counterfactual conditionals. These verbs help indicate the spatial frame of the conditional, which can influence the interpretation of the statement as counterfactual [26,27]. Events described with *qu* (go) are seen as more plausible than those using *lai* (come). This is because *qu* (go) takes on a non-literal meaning of prospective orientation, encoding future directionality that increases the perceived potentiality of the event. *Lai* (come) expresses a non-literal meaning of retrospective orientation, encoding a past direction that reduces plausibility. The combined use of directional verbs and temporal indicators could help readers distinguish between counterfactual conditionals and hypothetical conditionals, such as in Examples 2 and 3 [28]. However, it still remains unclear whether different directional verbs (e.g., *lai* [come] vs. *qu* [go]) could have distinct effects on the understanding of counterfactuals. 

While antecedent falsity is a key distinguishing feature between counterfactual and hypothetical conditionals, the implied causal relationship between the antecedent and consequent is also crucial for understanding counterfactual and hypothetical conditionals [13,29,30]. The implied causal relationship in counterfactual conditionals is particularly complex, as it involves the stated scenario and its negation in reality [13]. A critical yet underexplored aspect of this process is how lexical–semantic associations contribute to the formation and strength of these implied causal links. For instance, in the counterfactual “If I had studied harder yesterday, I would have passed the exam”, readers must process both the stated causal link (studying leads to passing) and the implied real-world outcome (not studying enough led to failing). The strength and plausibility of this causal relationship can significantly influence how readers interpret and process the conditional statement. Lexical–semantic relationships play a crucial role in constructing and reinforcing these causal links. The semantic proximity between “studying” and “passing” in the aforementioned example helps readers quickly establish the causal connection [29,30]. This process of semantic facilitation may differ between counterfactual and hypothetical conditionals due to the additional cognitive demand of processing the negated real-world scenario in counterfactuals. To investigate these subtle differences in causal link formation between counterfactual and hypothetical conditionals, we propose using lexical–semantic analysis techniques such as latent semantic analysis (LSA) or *Word2Vec* models [31,32]. 

Despite these insights, there is still a lack of psycholinguistic evidence examining how these lexical pragmatic factors collectively impact the activation and processing of Chinese counterfactual and hypothetical conditionals. Moreover, it remains unclear whether readers process these lexical-pragmatic cues differently in counterfactual conditionals than in hypothetical conditionals. Further research is needed to elucidate how these cues interact and influence the strength of antecedent falsity and their impact on the comprehension of different types of conditionals in Mandarin Chinese. To address this issue, the current study tried to determine if readers are sensitive to specific elements and identify the processing course when readers distinguish the hypothetical and counterfactual conditional sentences.

### 1.2. The Present Study

While previous studies in languages like German have provided valuable insights into counterfactual and hypothetical conditional processing [6], the findings from these studies may not be fully generalizable to languages with different linguistic structures, such as Mandarin Chinese [33]. Moreover, a long debate has existed about whether Chinese speakers have weaker counterfactual reasoning abilities than some Indo-European speakers since Chinese lacks counterfactual morphosyntactic markers [20,34]. Thus, the mechanisms by which pragmatic factors influence conditional comprehension may differ significantly from those observed in Indo-European languages. Therefore, conducting research in Mandarin Chinese is crucial for establishing the universality or specificity of cognitive mechanisms involved in conditional reasoning. This study addressed the extent to which manipulating pragmatic factors influences readers’ understanding and ability to distinguish between counterfactual and hypothetical conditional sentences in Mandarin Chinese. We manipulated different conditional types by changing temporal indicators in the antecedent, with a future temporal indicator (e.g., *mingtian* [tomorrow]) constructing hypothetical conditionals and a past temporal indicator (e.g., *zuotian* [yesterday]) constructing counterfactual conditionals. To detect whether readers could successfully infer the counterfactual or hypothetical meaning, we also manipulated the consistency of described events with world knowledge, which was determined by the existence of a negator in the subordinate clause. Participants were required to use their real-world knowledge to detect whether it is consistent (e.g., “*Ruguo zuotian buxiayu, xiaoliu jiuqu gongyuan wanhuati*” [“If it had not rained yesterday, Xiaoliu would have gone to the park to play on the slide”]) or inconsistent (e.g., “*Ruguo zuotain xiayu, xiaoliu jiuqu gongyuan wanhuati*” [“If it rained yesterday, Xiaoliu would have gone to the park to play on the slide”]). In order to fit with Chinese participants’ reading habits, the negator and temporal indicator appeared in the subordinate clause of the conditionals, and the directional verb appeared in the main clause of the conditionals since readers can fully understand the consistency when they finish comprehending the whole conditionals based on their factual world knowledge. 

There were four hypotheses. First, if comprehending the logical relationship between clauses is influenced by the temporal order of the two events described within conditional sentences, the comprehensibility score of conditionals with the temporal indicator that describes the future (hypothetical conditionals) would be higher than those with the past time-oriented temporal indicator (counterfactual conditionals). Second, if semantic inconsistency between main and subordinate clauses decreases the perceived probability of the event pairs described, the comprehension score should be lower for conditional sentences that contain inconsistent event pairs clashing with real-world knowledge compared to sentences with consistent events. Thirdly, we also predicted that events described with the directional verb *lai* (come) would be less comprehensible compared to those described with the directional verb *qu* (go). Fourthly, if the lexical–semantic relationship between main and subordinate clauses could impact comprehension, the semantic similarity across clauses would show a greater level of similarity for hypothetical over counterfactual conditionals.

## 2. Materials and Methods

### 2.1. Participants

Twenty-four native Mandarin-Chinese speakers were recruited (20 females; mean age = 22.75 ± 2.12 years) for the comprehension task. The sample size was calculated using G.power 3.1.9.2 [35], with an alpha level of 0.05. The power analysis showed that at least 15 participants were required for the main effect of consistency to reach the power of 95%. Given that the actual number of the current study (*n* = 24) was more extensive than this estimation, statistical power was ensured. All participants were right-handed undergraduate students, and none experienced psychiatric or neurological illness. Before the experiment, they all received and signed the informed consent. This study was approved by the local Ethics Committee.

### 2.2. Design, Materials, and Procedure

Two hundred quadruplets of complex sentences were developed as the critical stimuli (see Table 1 for the sentence exemplar). Each sentence was a Chinese conditional containing a main clause and subordinate clause. We maintained the main clauses across different conditions, whereas the differences existed only in the subordinate clause, where the temporal indicators and negator were manipulated. In Table 1, *mingtian* (tomorrow) in example A was the temporal indicator indicating the future (hypothetical conditionals), and *zuotian* (yesterday) in example C was the temporal indicator indicating the past (counterfactual conditionals). Antecedent polarity refers to the positive or negative framing of events described in the antecedent and consequent. The presence or absence of the negator *bu* (no) in the subordinate clause determined the antecedent polarity. Sentences with the negator *bu* (e.g., examples A and C in Table 1) had negative antecedent polarity, while those without *bu* (e.g., examples B and D) had positive antecedent polarity. The presence or absence of the negator *bu* (no) in the subordinate clause determines both the antecedent polarity and the consistency of the sentence. Specifically, sentences with the negator have negative antecedent polarity and are consistent, while those without the negator have positive antecedent polarity and are inconsistent. In the comprehension rating task, participants were asked to judge the consistency of the sentences. While the comprehension rating task focused on participants’ explicit judgments of sentence consistency, the semantic similarity analysis allowed us to examine the more subtle effects of antecedent polarity on semantic processing. This dual-method approach provided a more comprehensive understanding of how our manipulations affected conditional sentence processing. In addition, the directional verbs were either *lai* (come) or *qu* (go), manipulated as a between-item variable, with half of the materials containing *lai* and the other half containing *qu*.

Participants needed to rate the comprehensibility of each sentence on a 7-point Likert scale, where 1 indicated completely incomprehensible, and 7 indicated completely comprehensible. They were instructed to read each sentence carefully and use their real-world knowledge to judge how understandable the logical relationship between the main clause and subordinate clause was.

### 2.3. Statistical Analysis

The statistical models were built on the comprehensibility and the semantic similarity values. In this study, we utilized *Word2Vec* word embeddings to compute semantic similarity between sentences and within subordinate clauses. *Word2Vec* is a neural network-based approach for learning distributed vector representations of words based on their contextual information. By directly modelling the semantic content through modern word embeddings, this approach can quantify semantic similarity more accurately compared to traditional latent semantic analysis [36]. We trained *Word2Vec* models on a large-scale Chinese corpus using the *Gensim* library in Python [37]. The resulting *Word2Vec* model provides a dense vector representation for each word, with semantically similar words having similar vector representations. Prior to computing semantic similarity, the texts were preprocessed. Specifically, conditional connectives such as *ruguo…jiu* (if...then) were removed to isolate the content of main and subordinate clauses. The remaining clauses were segmented into words as the basic units for subsequent analysis. Additionally, stop words like punctuation were removed, retaining only content words in the sentences. Notably, the negator *bu* (no) was not separately segmented. This preprocessing allowed the following semantic similarity calculation to focus solely on the semantic information of the content words in the relevant parts of the sentences, avoiding potential interference from conditional markers. After preprocessing, the main and subordinate clauses were input into the trained *Word2Vec* model to derive a vector representation for each word. Cosine similarity between the clause vectors was then calculated as the semantic similarity between the main and subordinate clauses. Meanwhile, the cosine similarity of vectors within the subordinate clauses was also computed as the semantic similarity within the subordinate clauses. 

#### 2.3.1. Semantic Similarity Calculation

The semantic similarity analysis was conducted for two primary purposes. First, the semantic similarity allowed us to directly examine whether using negators and different directional verbs produced differences in lexical–semantic co-occurrence under different conditional types triggered by different temporal indicators (past vs. future). Second, including semantic similarity factors as controls in subsequent comprehensibility linear mixed-effects models enabled isolating the unique impacts of these pragmatic variables beyond any influence from lexical–semantic associations. To determine the effects of the temporal indicator, antecedent polarity, and directional verb (between item) and their interactions on semantic similarity scores, two linear mixed-effects models (LMEM) were built. One semantic similarity model (a) was specified for analyzing lexical–semantic associations within the subordinate clause. Based on a model-selection procedure with the likelihood ratio test [38], the full model including the fixed effects of the temporal indicator and antecedent polarity and their interactions were included in the best-fitting model for analyzing the semantic similarity value. A second semantic similarity model (b) was specified for analyzing the lexical–semantic associations across the main and subordinate clauses. This model included fixed effects for the temporal indicator, consistency, and directional verb and their interactions. The within- and across-clause semantic similarity models aimed to separately quantify the impacts of lexical–semantic co-occurrence of the independent variables within the subordinate clause vs. between the main and subordinate clauses.

**Model** **a.** *Semantic similarity (within clauses) ~ Temporal Indicator × Antecedent Polarity + (1|Item)*

**Model** **b.** *Semantic similarity (across clauses) ~ Temporal Indicator × Antecedent Consistency × Directional Verb + (1|Item)*

The lme4 [39] and lmerTest packages [40] of R-studio (Version 3.1.0, http://cran.r-project.org, accessed on 6 May 2023) were used for all statistical analyses. 

#### 2.3.2. Comprehensibility

To determine the effects of the temporal indicator, consistency (since the existence of a negator (*meiyou* [there is not]) determines the consistency, this study only maintains the effect of consistency in statistics analysis of comprehensibility), and directional verb and their interactions on comprehensibility scores, two linear mixed-effects models (LMEM) were built. Model c and model d both focused on temporal indicators, as the manipulation of different types of conditionals relies on temporal indicator differences. However, the two models differed in whether they included consistency and directional verb factors or semantic similarity factors in order to isolate their distinct influences beyond temporal indicators. The model-selection procedure of the first LMEM model c started with a baseline model including subjects and items as random intercept [41,42]. Parsimonious models were determined by AIC (Akaike Information Criterion) in a stepwise algorithm using the step function from the stats package and visually inspected for no obvious deviations from homoscedasticity or normality through residual plots. The VIF (Variance Inflation Factor) of all predictor variables was less than 10 [43] (see Table 1). Compared to the baseline model, the best-fitting models were identified:

**Model** **c.** *Comprehensibility ~ Temporal Indicator × Consistency × Directional Verb + Semantic Similarity (across clauses) + Semantic Similarity (within clauses) + (1|Subject) + (1|Item)*

In order to clarify whether the semantic similarity (across clauses) has an impact on the temporal indicator, we tested model d, which also includes subjects and items as the baseline model. Parsimonious models were determined by AIC in a stepwise algorithm using the step function from the stats package and visually inspected for no obvious deviations from homoscedasticity or normality through residual plots. The VIF of all predictor variables was less than 10 (see Table 2 and Table 3). Compared to the baseline model, the best-fitting model was identified:

**Model** **d.** *Comprehensibility ~ Temporal Indicator × Semantic Similarity (across clause) +Semantic Similarity (within clauses) + (1|Subject) + (1|Item)*

**Table 2:** The Variance Inflation Factor (VIF) of all predictors in model c.

Variables	VIF
Temporal Indicator	4.04
Consistency	4.00
Directional Verb	2.41
Semantic Similarity (within clause)	1.03
Semantic Similarity (across clause)	1.04
Temporal Indicator × Consistency	6.00
Temporal Indicator × Directional Verb	4.92
Consistency × Directional Verb	4.92
Temporal Indicator × Consistency × Directional Verb	6.46

**Table 3:** The Variance Inflation Factor (VIF) of all predictors in model d.

Variables	VIF
Temporal Indicator	1.64
Semantic Similarity (within clause)	1.01
Semantic Similarity (across clause)	1.95
Temporal Indicator × Semantic Similarity (across clause)	2.52

## 3. Results

### 3.1. Semantic Similarity Analysis

**Figure 1:**
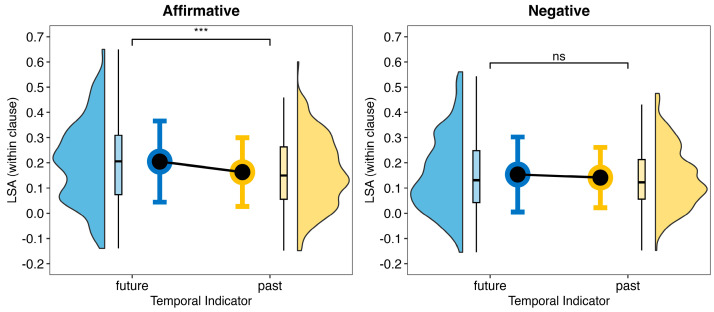
Semantic similarity (within clause) score of the antecedent polarity and temporal indicator. Blue represents the LSA values with a temporal indicator of the future, while yellow represents the LSA values with a temporal indicator of the past. The significant threshold is represented by the number of symbols (*** *p* < 0.001, ns *p* ≥ 0.05).

Model a showed a significant main effect of antecedent polarity: *F* (1, 199) = 61.18, *p* < 0.001, η^2^ = 0.24. The semantic similarity value (within the clause) was higher in affirmative conditions (0.18) than in negative conditions (0.15). The model also showed a main effect of the temporal indicator: *F* (1, 199) = 15.81, *p* < 0.001, η^2^ = 0.07. The semantic similarity value was higher in antecedents that contained the future temporal indicator than those that contained the past temporal indicator (see Figure 1).

**Figure 2:**
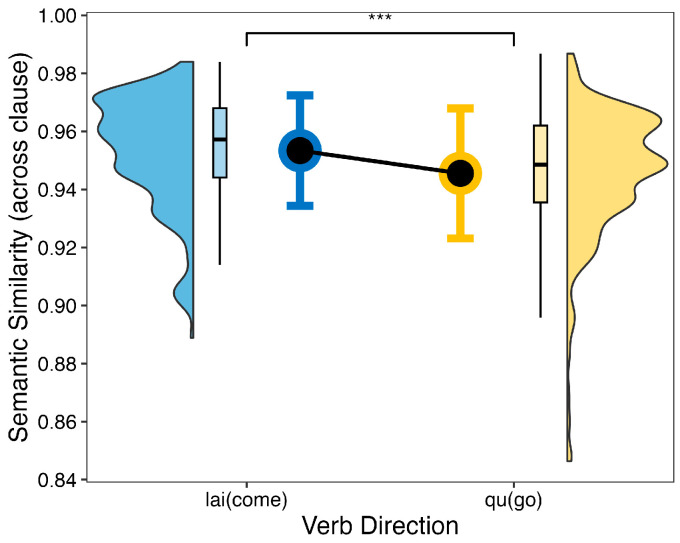
Semantic similarity (across clause) score of the directional verb. Blue represents the semantic similarity values with the verb direction *lai* (come), while yellow represents the semantic similarity values with the verb direction *qu*(go). The significant threshold is represented by the number of symbols (*** *p* < 0.001).

Model b revealed that the effect of directional verb was significant: *F* (1, 199) = 7.37, *p* = 0.007, η^2^ = 0.05. The semantic similarity value was higher in the sentence with *lai* (come) than with *qu* (go) (see Figure 2). However, neither temporal indicator (*F* (1, 199) = 1.26, *p* = 0.26, η^2^ < 0.001) nor consistency was significant (*F* (1, 199) = 1.08, *p* = 0.30, η^2^ < 0.001).

### 3.2. Comprehensibility

**Figure 3:**
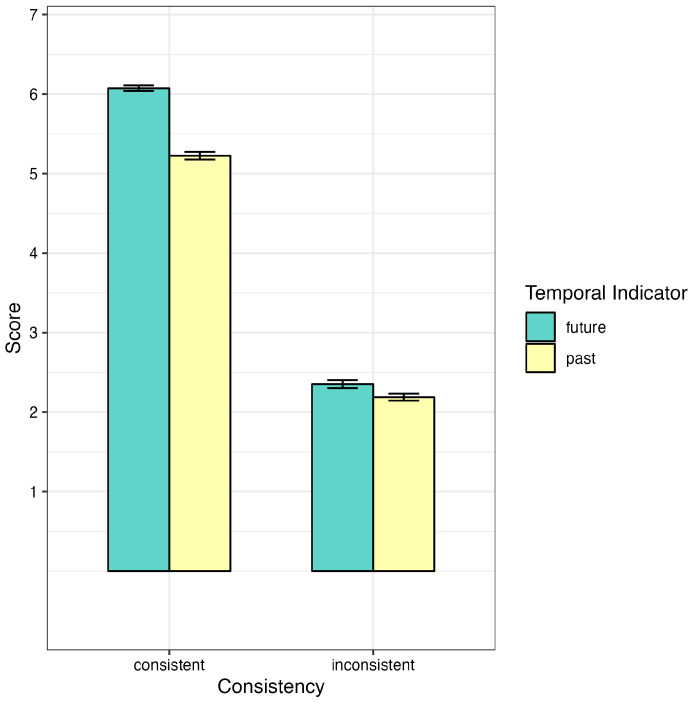
Comprehensibility of consistency and temporal indicator. Mean and standard error per condition were plotted.

Model c clearly showed the significant main effects of the temporal indicator (*F* (1, 29) = 33.70, *p* < 0.001, η^2^ = 0.54) and consistency (*F* (1, 29) = 41.21, *p* < 0.001, η^2^ = 0.92). The comprehensibility score was higher in the consistent condition (M = 5.65, SD = 1.55) than in the inconsistent condition (M = 2.27, SD = 1.61). Moreover, the conditional sentences of the future temporal indicator (M = 4.21, SD = 2.38) were higher than those of the past temporal indicator in the antecedent (M = 3.71, SD = 2.21). The interaction of the temporal indicator and consistency was also significant: *F* (1, 4579) = 65.09, *p* < 0.0001, η^2^ = 0.01. The follow-up analysis revealed that sentences of the future temporal indicator were significantly more comprehensible than those of the past temporal indicator when the antecedent and the consequent were logically consistent (*β* = 0.85, *SE* = 0.09, *z* = 8.98, *p* < 0.0001, 95% CI: [0.66, 1.03]), whereas the contrast between temporal indicators under the inconsistent condition did not reach any significance (*β* = 0.16, *SE* = 0.09, *z* = 1.74, *p* = 0.08, 95% CI: [−0.02, 0.35]) (see Figure 3). In addition, the more increased comprehensibility score in the consistent than the inconsistent sentences was higher in those that contained past (*β* = 3.04, *SE* = 0.06, *z* = 1.10, *p* < 0.001, 95% CI: [2.92, 3.16]) compared to those with future temporal indicators (*β* = 0.72, *SE* = 0.50, *z* = 2.50, *p* < 0.001, 95% CI: [3.60, 3.83]). The interaction effect between consistency and directional verb was also significant: *F* (1, 4579) = 3.89, *p* = 0.04, η^2^ < 0.001. The follow-up analysis demonstrated that under consistent conditions, *lai* (come) had a lower comprehensibility score than *qu* (go) (*β* = 0.17, *SE* = 0.08, *z* = −2.24, *p* = 0.02, 95% CI: [−0.32, −0.02]), whereas the contrast between *lai* and *qu* under inconsistent conditions was not significant: *β* = −0.01, *SE* = 0.08, *z* = −0.06, *p* = 0.95, 95% CI: [−0.15, 0.14]. 

In model d, the effect of the temporal indicator was significant: *F* (1, 103) = 4.92, *p* = 0.03, η^2^ = 0.05. Sentences that contained future temporal indicators (4.21) had higher comprehensibility than sentences that contained past temporal indicators (3.70). The main effect of the semantic similarity (across clauses) was insignificant: *F* (1, 53) = 0.07, *p* = 0.80, η^2^ < 0.001. These results demonstrated that changes in temporal indicators could directly impact comprehensibility, while cross-clausal lexical–semantic association did not. This highlights the key role of temporal indicators in driving probability hierarchy effects during conditional comprehension.

## 4. Discussion

The current study aimed to investigate whether pragmatic factors differentially influence readers’ comprehension and distinction between counterfactual and hypothetical conditional sentences in Mandarin Chinese. The results of the comprehensibility clearly showed the following: (1) Readers could detect different temporal indicators when they comprehended conditional sentences. (2) When the event pairs across clauses were consistent with the reader’s real-world knowledge, participants reported a higher level of understandability regarding the logical relationship between the main clause and the subordinate clause. Conversely, inconsistency in these event pairs led to a lower reported understandability of this logical relationship. (3) The results also revealed differences based on the directional verb—under consistent conditions, *lai* (come) led to lower comprehensibility compared to *qu* (go). The semantic similarity analysis within the subordinate clause (model *a*) showed that the semantic similarity was higher in the antecedent with a future temporal indicator than in the clause where a past temporal indicator was included and was lower in the affirmative conditions than in the negative conditions. The semantic similarity results across clauses revealed that the semantic similarity between the main clause and subordinate clause of Chinese counterfactuals was stronger for the directional verb *lai* (come) than *qu* (go).

### 4.1. The Real-World Knowledge Influences the Activation of Different Conditional Sentences

Our current results showed that the temporal indicator that described the future was more comprehensible than the temporal indicator that indicated the past. The greater comprehensibility of future-oriented conditionals can thus be attributed to the reduced cognitive load associated with processing antecedent falsity. When readers encounter a future-oriented conditional, they can focus primarily on understanding the proposed causal relationship without the additional burden of reconciling this relationship with a contradictory known reality. This aligns with the nature of hypothetical conditionals, which primarily activate possible world scenarios without strongly implicating the falsity of the antecedent. In contrast, past-oriented conditionals, by strongly establishing antecedent falsity, require readers to engage in more complex cognitive processing. This involves not only understanding the proposed causal relationship but also maintaining awareness of its falsity in relation to known past events. This additional layer of processing may account for the observed lower comprehensibility of past-oriented conditionals in our study. These findings underscore the crucial role that temporal indicators play in modulating the strength of antecedent falsity in Mandarin Chinese conditionals. By influencing the degree of antecedent falsity, temporal indicators significantly impact the cognitive processes involved in conditional comprehension, leading to observable differences in comprehensibility between future-oriented (typically hypothetical) and past-oriented (typically counterfactual) conditionals. 

In analyzing the interaction effect of temporal indicators and consistency, we only found the difference of temporal indicators in the consistent conditions. This could be attributed to comprehenders being able to identify differences between hypothetical and counterfactual conditionals, primarily when the event pair across clauses aligned with real-world plausibility. The inconsistent scenarios contained implausible events (rainy day—go to the park to play the slide) contradicting real-world norms, which could obscure differences between conditional types. However, when event pairs formed a logically coherent sequence based on real-world norms, the different temporal indicators could effectively signal hypothetical vs. counterfactual meaning. Overall, the results could highlight how semantic congruency facilitates probability distinctions between conditional types during comprehension.

The interaction analysis of directional verbs and consistency also showed that only under consistent conditions does *lai* (come) have a lower comprehensibility score than *qu* (go). These observations can be attributed to the fact that the past-implying verb *lai* (come) may subtly decrease plausibility even in coherent scenarios, while the future-implying *qu* (go) maintains higher plausibility [26,27]. In particular, when real-world congruency provides a strong constraint, the differences between *lai* (come) and *qu* (go) in forming a coherent representation can emerge. This interaction could demonstrate how lexical–semantic and pragmatic factors can work in concert to signal plausibility distinctions in conditionals.

### 4.2. The Role of Semantic Similarity in Impacting Counterfactual Comprehension

The current semantic similarity results within the subordinate clause demonstrate that temporal indicators can influence counterfactual comprehension by modifying lexical–semantic co-occurrence within the subordinate clause. The hypothetical conditionals that contain future temporal indicators appear to have a stronger lexical–semantic association within the clause than the counterfactual conditionals that include the past temporal indicator. In addition, the comprehensibility scores follow the same pattern, with hypothetical conditionals being rated as more understandable than counterfactual conditionals. Thus, we assume that lexical–semantic association within the subordinate clause could be a key mechanism by which temporal indicators influence the understanding of counterfactual conditionals versus hypothetical conditionals.

The semantic similarity analysis across clauses found a significant effect of directional verbs, with *lai* (come) increasing semantic association more than *qu* (go). However, directional verbs did not impact comprehensibility. These results could be attributed to the dynamic interplay between pragmatic inference and lexical–semantic co-occurrence. Previous relevant studies have proposed that lexical–semantic associations could temporarily override pragmatic licensing or inferencing [44,45]. On the contrary, other relevant researchers have argued that pragmatic inferencing dominates the processing even when lexical–semantic relationships between individual words are well matched with each other [46,47]. The directional verbs of the current study are consistent with the latter account. Our study found that directional verbs did not have a significant impact on comprehension despite increasing semantic association between clauses based on semantic similarity. This suggests readers do not mainly rely on the lexical–semantic relationships of directional verbs when understanding hypothetical versus counterfactual conditionals. 

Overall, readers appear to focus more on other factors like temporal indicators and negators during conditional comprehension processing. The lexical–semantic associations of directional verbs may not contribute substantially to distinguishing hypothetical and counterfactual conditionals. 

### 4.3. The Mental Model Activation between Counterfactual and Hypothetical Conditionals

What cognitive mechanism enables comprehenders to activate different mental models between counterfactual and hypothetical conditionals? It is based on the previous literature that the ability to make accurate pragmatic inferences grounded in assessments of truth value and integration of real-world knowledge with the unfolding of the counterfactuals appears fundamental [6,7,48,49]. Moreover, previous studies on individual differences in counterfactual processing have also shown the role of pragmatic inferencing ability (e.g., social-communicative ability, literature-reading ability). For example, individuals with better social communicative pragmatics as shown by higher scores on the Autism Quotient Communication subscale were less swayed by knowledge-based expectations when processing counterfactuals [50] or extracting pragmatic information from the discourse markers [51]. This highlights how social-communicative abilities modulate pragmatic reasoning. Additionally, higher literature exposure enhances counterfactual pragmatic inference, with more reading experience associated with a greater inclination for elaborative inference [5]. This literature-driven cognitive flexibility may allow more complex reanalysis of conditionals. Together, such findings suggest comprehension proficiency fosters greater sensitivity to the pragmatic nuances differentiating hypothetical and counterfactual meaning. In the current study, the pragmatic inference process could be disrupted under inconsistent conditions. Processing differences between hypothetical conditionals with future temporal indicators and counterfactual conditionals with past indicators only emerged in conditions where the truth value can be clearly verified based on events plausibility (e.g., did not rain—go to the park to play the slide). When the actual presented events are obscured by inconsistencies with real-world constraints, comprehenders struggle to integrate sentences with world knowledge to make pragmatic probability assessments. 

Previous research on examining the activation of different mental models between counterfactual and hypothetical conditionals focused on morphologically rich languages (such as English, German, etc.), where comprehenders can leverage syntactic markers like past tense and subjunctive mood in “If P, then Q” structures to make pragmatic inferences [15]. This “syntactic-driven” differentiation may be less applicable to Mandarin Chinese, where hypotheticals and counterfactuals share structural forms. However, the current findings showcase that lexical markers such as temporal indicators and directional verbs can modulate conditional processing and supply additional evidence that extends Comrie’s theoretical models by showing the pragmatic constraints on differentiating the mental model activation between counterfactual and hypothetical sentences in languages where lexical information could be an important cue. This finding could open avenues for future research to directly compare the processing mechanisms underlying syntactically versus lexically driven probability hierarchies across languages. 

Our current findings on comprehensibility ratings and semantic similarity analyses in Mandarin Chinese conditionals provide a solid foundation for future online studies examining mental model activation. However, it is important to acknowledge that our study does not address the neural mechanisms underlying mental model activation directly. Therefore, future online studies (e.g., EEG or MEG) could utilize our comprehensibility ratings to select well-matched counterfactual and hypothetical conditionals. Incorporating our semantic similarity measures to create carefully controlled stimulus sets allows examination of how semantic relatedness interacts with conditional type in neural processing. If future studies find larger N400/M350 amplitudes and stronger theta band activity for counterfactuals at critical time points (e.g., temporal indicator, final word of subordinate clause, etc.) [13,52,53], it may reflect an increased cognitive load of maintaining two mental representations. This approach may provide neural evidence for or against the single vs. dual mental model theories while elucidating how pragmatic factors and semantic relationships influence the online processing of these complex linguistic structures. 

## 5. Conclusions

The current study investigated whether pragmatic factors differentially influence readers’ comprehension and distinction between counterfactual and hypothetical conditional sentences in Mandarin Chinese. The findings demonstrate the following: (1) Temporal indicators impacted conditional processing partly through lexical–semantic co-occurrence, with future temporal indicators being more comprehensible than past temporal indicators in hypotheticals versus counterfactuals, respectively. (2) When the event pairs across clauses align with the reader’s real-world knowledge, the conditional meaning becomes more comprehensible, while inconsistency impedes this comprehension. (3) Readers do not predominantly depend on the lexical–semantic relationships of directional verbs in comprehending hypothetical versus counterfactual conditionals. (4) We assume that the cognitive mechanism distinguishing between counterfactual and hypothetical conditionals lies in pragmatic inference performance, which is grounded in truth value assessments and the integration of real-world knowledge.

## Figures and Tables

**Table 1:** Sentence exemplars in one set of experimental stimuli.

	Example Sentences
A	Ruguo mingtian buxiayu, liuxing jiu qugongyuan wanhuati. If it does not rain tomorrow, Liu Xing will go to the park to play the slide.

B	Ruguo mingtian xiayu, liuxing jiu qugongyuan wanhuati. If it rains tomorrow, Liu Xing will go to the park to play the slide.

C	Ruguo zuotian buxiayu, liuxing jiu qugongyuan wanhuati. If it had not rained yesterday, Liu Xing would have gone to the park to play the slide.

D	ruguo zuotian xiayu, liuxing jiu qugongyuan wanhuati. If it rained yesterday, Liu Xing would have gone to the park to play the slide.


## Data Availability

The data presented in this study are available on request from the corresponding author. The data are not publicly available due to privacy.
